# Characteristics, treatment outcomes and experiences of COVID-19 patients under home-based care in Kapelebyong district in Uganda: a mixed-methods study

**DOI:** 10.1186/s41182-022-00486-5

**Published:** 2022-12-14

**Authors:** Eudu James, Benon Wanume, Milton W. Musaba, Ritah Nantale, Vivian Mutaki, Brendah Nambozo, David Okia, David Soita, Agnes Napyo, Joseph K. B. Matovu, Racheal Namulondo, Jovani Lubaale, Francis Okello, Ronald Mulebeke, Abel Kakuru, Nancy Amejje, David Emojong, Charles Okolimong, Simple Ouma, Sam Okware, Peter Olupot-Olupot, David Mukunya

**Affiliations:** 1 Department of Community and Public Health, Busitema University, Mbale, Uganda; 2 Department of Obstetrics and Gynecology, Busitema University, Mbale, Uganda; 3 Department of Nursing, Busitema University, Mbale, Uganda; 4 Department of Disease Control and Environmental Health, Makerere University School of Public Health, Kampala, Uganda; 5 Department of Research, The AIDs Support Organization, Mbale, Uganda; 6 Department of Research, Uganda National Health Research Organizations, Kampala, Uganda; 7 Department of Research, Mbale Clinical Research Institute, Mbale, Uganda; 8 Department of Research, Nikao Medical Center, Kampala, Uganda

**Keywords:** Home-based care, COVID-19, Uganda, Treatment outcome, Stigma, SARS-COV-2

## Abstract

**Background:**

A rapid increase in community transmission of COVID-19 across the country overwhelmed Uganda’s health care system. In response, the Ministry of Health adopted the home-based care strategy for COVID-19 patients with mild-to-moderate disease. We determined the characteristics, treatment outcomes and experiences of COVID-19 patients under home-based care during the second wave in Kapelebyong district, in eastern Uganda.

**Methods:**

We conducted a sequential explanatory mixed-methods study. We first collected quantitative data using an interviewer-administered questionnaire to determine characteristics and treatment outcomes of COVID-19 patients under home-based care. Cured at home was coded as 1 (considered a good outcome) while being admitted to a health facility and/or dying were coded as 0 (considered poor outcomes). Thereafter, we conducted 11 in-depth interviews to explore the experiences of COVID-19 patients under home-based care. Multivariable logistic regression was used to assess factors associated with poor treatment outcomes using Stata v.15.0. Thematic content analysis was used to explore lived experiences of COVID-19 patients under home-based care using NVivo 12.0.0

**Results:**

A total of 303 study participants were included. The mean age ± standard deviation of participants was 32.2 years ± 19.9. Majority of the participants [96.0% (289/303)] cured at home, 3.3% (10/303) were admitted to a health facility and 0.7% (2/303) died. Patients above 60 years of age had 17.4 times the odds of having poor treatment outcomes compared to those below 60 years of age (adjusted odds ratio (AOR): 17.4; 95% CI: 2.2–137.6). Patients who spent more than one month under home-based care had 15.3 times the odds of having poor treatment outcomes compared to those that spent less than one month (AOR: 15.3; 95% CI: 1.6–145.7). From the qualitative interviews, participants identified stigma, fear, anxiety, rejection, not being followed up by health workers and economic loss as negative experiences encountered during home-based care. Positive lived experiences included closeness to friends and family, more freedom, and easy access to food.

**Conclusion:**

Home-based care of COVID-19 was operational in eastern Uganda. Older age (> 60 years) and prolonged illness (> 1 months) were associated with poor treatment outcomes. Social support was an impetus for home-based care.

## Background

Uganda experienced the second wave of COVID-19 between April and September 2021. The proportion of patients with severe disease in the second wave was twice that observed in the first wave, which overwhelmed the Ugandan health system [1]. Due to this, the ministry of health switched from the strategy of health facility isolation of all COVID-19 confirmed patients to home-based care strategy [2]. Under the home-based care strategy, mild-to-moderate COVID-19 patients are provided with the required care at the patient’s residence by a care giver who may be a family member, a friend or a member of the local community while cooperating with the advice and support from trained health workers [3]. This was consistent with the World Health Organization (WHO) recommendations for home-based care for newly confirmed or suspect COVID-19 patients having no symptoms or having mild illness [3].

The high population density, poor housing, overcrowding, poor infrastructure and inadequate access to water and sanitation in many sub-Saharan communities became major constraints for effective home-based care of COVID-19 patients [2]. Furthermore, the unique socio-cultural context, beliefs and stigma associated with life threatening infectious diseases on the continent is a challenge to home-based care [2].

Despite evidence of successful home-based care for management of COVID-19 in many European countries [4, 5], there is limited information on the characteristics, treatment outcomes and experiences of COVID-19 patients under home-based care in sub-Saharan Africa. This information could be used to develop evidenced-based recommendations for home-based care of COVID-19 patients in sub-Saharan Africa. This study determined the characteristics, treatment outcomes and lived experiences of COVID-19 patients under home-based care in Kapelebyong district in eastern Uganda.

## Methods

### Study design

We conducted a sequential explanatory mixed-methods study, where quantitative data were collected and analyzed first. This was later followed by qualitative data, which was collected to better understand the experiences of participants under COVID-19 home-based care.

### Study setting

The study was conducted between November 2021 and February 2022 in Kapelebyong district in the eastern region of Uganda bordered by Napak district to the north, Katakwi district to the east, Amuria district to the south, Alebtong district to the west and Abim district to the north-west. Kapelebyong has a total population of 168,242 people. Of these, 94,578 (56.2%) are female while 73,664 (43.8%) are male*.* Kapelebyong district has one constituency of Kapelebyong, 11 sub-county level administrative units, 55 parish level administrative units and 327 villages. The district has the district task force and the sub-county task force for coordination of COVID-19 management.

Kapelebyong has 14 health facilities; 1 health center (HC) 4, 3 HC3s (2 Government, 1 private not for profit), 9 HC2s and 1 Nursing home which is private for profit. Services offered include; out patients’ department, maternal and child health, laboratory services, HIV Services, family planning and theater services for only Kapelebyong HC4 with a bed capacity of 85. Health workers from HC3s and HC4 were trained and equipped with knowledge on diagnosis and management of COVID-19 patients under home-based care. Only patients that did not require admission at the time of diagnosis based on a clinician’s assessment were put under home-based care. All COVID-19 patients that needed admission were referred to Soroti regional referral hospital.

### Study population

All COVID-19 patients under home-based care in Kapelebyong district were eligible for this study. COVID-19 patients of all age groups, both male and female diagnosed using a PCR Test or a rapid diagnostic test, put under home-based care in Kapelebyong district and gave informed consent were included in this study. We excluded COVID-19 patients who were too sick to talk or those with severe mental disability.

### Quantitative component

#### Sample size

We sampled all COVID-19 patients under home-based care in Kapelebyong district that met the inclusion criteria and this gave us a sample size of 303. This sample size results in an absolute precision of 1.6% to 5.6%, i.e., the difference between the point estimate and the 95% confidence interval (CI) for prevalence values of poor outcomes ranging from 2 to 50%.

#### Sampling frame

We used the District Health Office database and health facility data base with locator information of all COVID-19 Patients under home-based care in Kapelebyong district. The district COVID-19 home-based care data are stored in the District Health Information Software 2 **(**DHIS2) tool. This data was cross-checked to ensure consistency with data at the health facilities stored at the facility COVID-19 home-based care registers. The facility COVID-19 home-based care registers capture identification and location information of all COVID-19 patients under home-based care within their catchment area such as; patients age, sex, date of COVID-19 test, date enrolled onto home-based care, place of residence, telephone number among others. The health facilities also had the list of all community health workers attached to each of these health facilities and their contacts. This information enabled us access all the COVID-19 patients under home-based care in Kapelebyong district. Additionally, the lead researcher in this study was the district focal person for home-based care services in Kapelebyong and played a key role in coordinating COVID-19 home-based care activities, so there was no challenge in accessing and enrolling participants into the study.

#### Study variables

The dependent variable was treatment outcomes of COVID-19 patients under home-based care. These were divided into good outcome used to define patients that cured at home and poor outcome used to define patients that were admitted to a health facility and/or died while under home care.

The independent variables were; socio-demographic factors (age, marital status, tribe, educational level, income levels, occupation, and religion), presence of comorbidities, vaccination status, number of vaccination doses, monthly family income, duration in care and follow up by health workers. We calculated wealth tertiles from an asset based index using principal component analysis. The following assets were considered: radio, television, mobile phone bicycle, motorcycle, car, computer, permanent house and piped water.

### Data collection

We used two trained research assistants to collect data electronically using an interviewer administered questionnaire designed in Kobo Toolbox (Cambridge, Massachusetts, USA). All participants were followed at their homes and face-to-face interviews were conducted in a secure environment that allowed free interaction between the participant and the interviewer after obtaining written informed consent from the participant. An abstraction tool prepared for the study was used to collect data about the deceased patients from their medical records.

### Statistical analysis

We summarized categorical variables as proportions and continuous variables as mean (standard deviation). We computed described analyses to determine the percentage of home-based care patients that cured, those that were eventually hospitalized, and those that were reported as dead at the time of interview. Dead patients were excluded from further analyses since they were not alive to be interviewed. We conducted multivariable logistic regression to determine the factors associated with poor treatment outcomes among COVID-19 patients under home-based care while controlling confounders. Factors with a p-value of less than 0.2 at bivariable analysis, and those known to affect treatment outcomes of COVID-19 patients from literature were included in the multivariable analysis. Adjusted odds ratios (AOR), 95% Confidence interval and p-values were calculated at a statistical significance at a *p*-value < 0.05. We used Stata V.15.0. (StataCorp LLC, College Station, Texas, United States of America) for analysis.

### Qualitative component

#### Participants’ selection

We purposively selected 11 participants among those that were part of the quantitative interviews to explore their experiences while under home-based care. Participants were followed at home.

#### Data collection and sampling

We collected qualitative data on the experiences of patients under home-based care in Kapelebyong district using an in-depth interview guide. An interviewer that is experienced in conducting qualitative interviews conducted the face-to-face in-depth interviews in a secure environment that allowed free interaction between the interviewer and the participant. Probing questions were used to get rich information on the issues that arose during the discussions. The interviews would take between 20 and 30 min. With permission from the participants, the interviews were audio recorded, transcribed verbatim and translated into English for those conducted in *Ateso.*

### Data analysis

We used thematic content analysis to analyze the data. The analysis followed a five-step process. First, we read through the transcripts and became familiar with the data. Secondly, we organized data in a meaningful way and generated the initial codes. Once the data had been sufficiently coded and saturation reached, we identified themes. We then reviewed and modified themes and put together all data relevant to each theme. Data were managed in NVivo 12.0.0 (QRS International, Cambridge, MA). Examples of meanings units, codes, categories and themes from qualitative content of interviews about experiences of COVID-19 patients under home-based care are shown in Table 1.

**Table 1:** Examples of meanings units, codes, categories and themes from qualitative content of interviews about experiences of COVID-19 patients under home-based care

Meaning units/quotes	Codes	Categories/issues	Themes
When my son's cough intensified, I called the doctor and they sent an ambulance to come and take him” [P11, 60 years female, IDI] When I got COVID-19, waking up became a problem because I had general body pains so it became a bit difficult. I would wake up at around 9-10 am and I wouldn't have energy to get out of bed” [P01, 35 years, female, IDI]	Symptoms of COVID-19	Participants argued that they experienced persistent and worsening intermittent signs and symptoms of the COVID-19 disease	Experiences of COVID-19 patients and survivors managed under home-based care
We all got shocked and what came in mind was death. People were dying. When you'd listen to the radio, we were all only hearing about people dying of COVID-19. We were in fear. We even lost sleep because we thought that we were going to die” [P11, 60 years female, IDI]	Fear and anxiety	Participants reported several forms of fear due to the misconception that when one gets COVID-19, they wouldn’t survive	
After two weeks, I went back to hospital to do a test to confirm if I had recovered and I found the laboratory closed. I was then sent out by a health worker saying that they didn't know if I had recovered or not. I felt segregated, lonely and isolated” [P01, 35 years female, IDI] There's no way I could move anywhere because when people see you, they would think you're spreading the disease. Even the pupils I was teaching, they could see me and run. They even nicknamed, "corona". One day I tried to move out because they'd told her to be doing exercise, when the children saw them, they started shouting, "corona, corona" and we decided to come back home” … [P11, 60 years, female, IDI]	Stigma	Participants reported having experienced stigma both at the health facilities and community, as they sought services from the health facilities and try to related with members in the community	

## Results

### Quantitative results

A total of 303 participants out of a population of 309 participants were interviewed. Since two of these participants had died, data on participant characteristics were only obtained from 301 participants. Majority [89.4% (269/301)] were aged less than 60 years, the mean age ± standard deviation was 32.2 ± 19.9. Slightly more than half of the participants were female [55.2% (166/301)] and were married [53.5% (161/301)]. Most of the participants [57.8% (174/301)] were Catholics and majority of them [87% (262/301)] were unemployed. Majority [65.9% (164/249)] had attained primary level of education and almost all of the participants [93.2% (275/295)] had not been vaccinated. [95.7% (288/301)] were *Itesots*. Other characteristics are shown in Tables 2 and 3.

**Table 2:** Socio-demographic characteristics of COVID-19 patients managed under home-based care in Kapelebyong district in eastern Uganda

Characteristic (n = 301)	Frequency (%)
*Age*	
< 60	269 (89.4)
≥ 60	32 (10.6)
*Sex*	
Male	135 (44.8)
Female	166 (55.2)
*Marital status*	
Single	112 (37.2)
Married	161 (53.5)
Separated/divorced	28 (9.3)
*Religion*	
Catholic	174 (57.8)
Anglican	100 (33.2)
Others	27 (9.0)
*Tribe*	
Iteso	288 (95.7)
Others	13 (4.3)
*Ever been to school*	
Yes	249 (82.7)
No	52 (17.3)
*Level of education*	
Primary	164 (65.9)
Secondary	57 (22.9)
Tertiary	28 (11.2)
*Employment status*	
Employed	39 (13.0)
Unemployed	262 (87.0)
*Monthly income*	
Less than 100,000 shillings	131 (43.5)
Between 100,000 and 500,000 shillings	128 (42.5)
Above 500,000 shillings	42 (14.0)
*Number of residential rooms in the house*	
< 3	102 (33.9)
≥ 3	199 (66.1)
*Do you share a residential room with someone*	
No	97 (32.2)
Yes	204 (67.8)

**Table 3:** Clinical characteristics of COVID-19 patients managed under home-based care in Kapelebyong district in eastern Uganda

Characteristic (*n* = 301)	Frequency (%)
*Anybody in the household been sick in the last 30 days*	
Yes	206 (68.4)
No	95 (31.6)
*Person suffered from (n* = *206)*	
Malaria	123 (59.7)
Cough	57 (27.7)
Flue	36 (17.5)
Pneumonia	8 (3.9)
Others	41 (19.9)
*Person sought medical advice*	
Yes	205 (99.5)
No	1 (0.5)
*Place of seeking medical advice (n* = *208)*	
Health facility	133 (63.9)
Clinic	95 (45.7)
From VHT	9 (95.7)
Others	2 (1.0)
*Reasons for COVID-19 testing (n* = *300)*	
Had signs of COVID-19	243 (81.0)
Family member had COVID-19	108 (36.0)
Was caring for a COVID-19 patient	9 (3.0)
Health worker told me to test	14 (4.7)
I just wanted to test	64 (21.3)
Others	35 (11.7)
*Feeling after being told was COVID-19 positive (n* = *300)*	
I thought I would die	143 (46.7)
Thought would be taken to the treatment center	21 (7.0)
Worried of what people would think	72 (24.0)
Thought would infect my family	104 (34.7)
Worried of family survival	64 (21.3)
Others	76 (25.3)
*Co-morbidities*	
Hypertension	21(7.1)
Diabetes	3 (1.0)
Heart problems	2 (0.7)
Asthma	6 (2.0)
HIV/AIDS	13 (4.4)
SCD	1 (0.3)
Chronic liver disease	1 (0.3)
Pregnancy	4 (1.3)
COPD	1 (0.3)
Tuberculosis	2 (0.7)
*Vaccinated*	
Yes	20 (6.8)
No	275 (93.2)
*Vaccine doses received*	
One	12 (63.2)
Two	7 (36.8)
*Duration of care*	
< 2 weeks	178 (59.1)
3 to 4 weeks	103 (34.2)
> 1 month	20 (6.6)
*Health workers follow-up*	
Yes	123 (41.1)
No	176 (58.9)
*Wealth tertile*	
Poorer	135 (44.9)
Middle	90 (29.9)
Richer	76 (25.2)

### Signs and symptoms experienced by COVID-19 patients under home-based care in Kapelebyong district in eastern Uganda

**Fig. 1:**
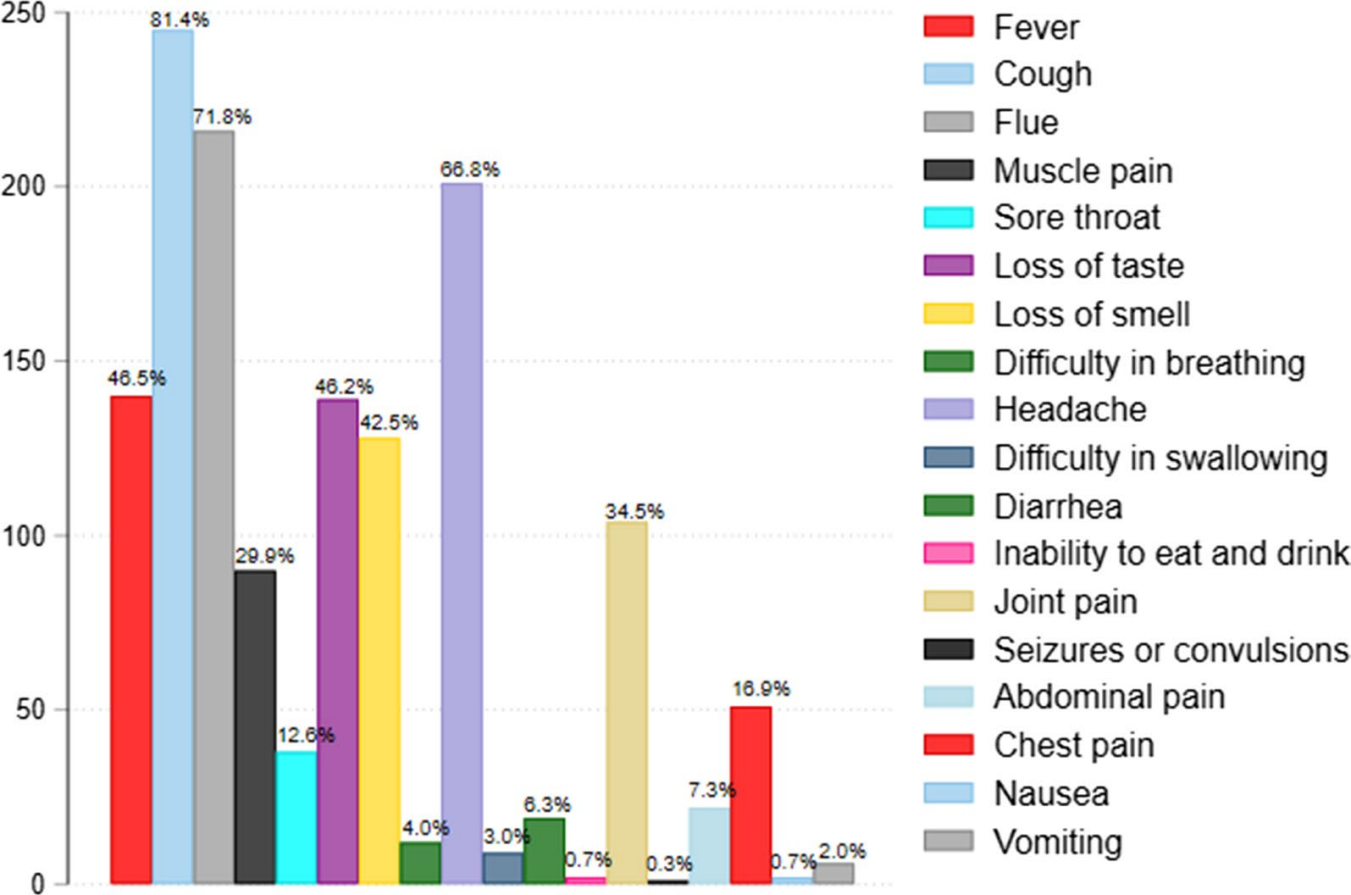
Signs and symptoms experienced by COVID-19 patients managed under home-based care in Kapelebyong district in eastern Uganda

Cough and flue were the most commonly cited symptoms [81.4% (245/301)] and [71.8% (216/301)], respectively. [66.8% (201/301)] and [46.5% (140/301)] experienced headache and fever, respectively. [46.2% (139/301)] experienced loss of taste, [42.5% (128/301)] lost sense of smell and [34.6% (104/301)] had joint pain. Others signs and symptoms are shown in Fig. 1.

### Treatment outcomes of COVID-19 patients managed under home-based care in Kapelebyong district in eastern Uganda

**Fig. 2:**
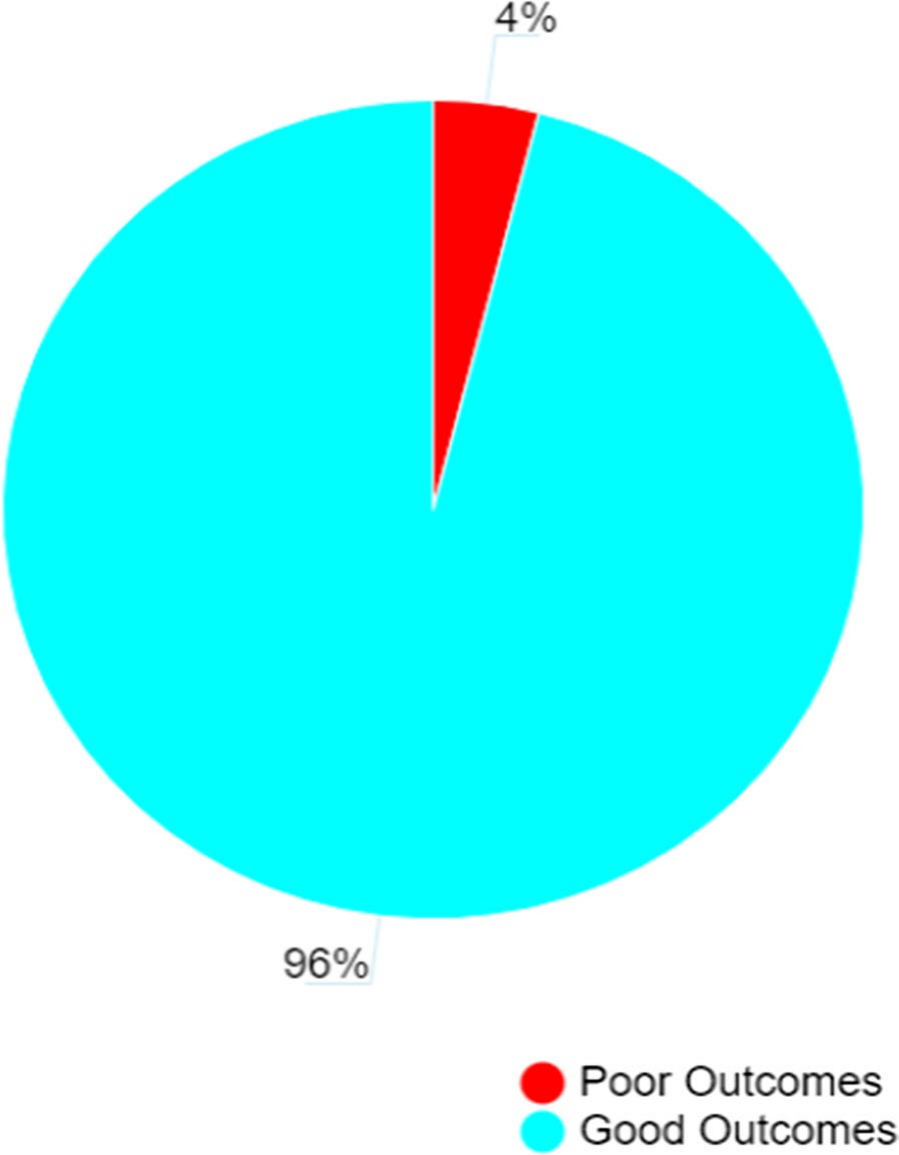
Treatment outcomes of COVID-19 patients managed under home-based care in Kapelebyong district in eastern Uganda

Of the 303 patients managed under home-based care in Kapelebyong district in eastern Uganda, majority [96.0% (291/303)] cured at home, [3.3% (10/303)] were admitted at the hospital and 0.7% (2/303) died. Cured at home was considered as a good outcome [96.0% (291/303)] while being admitted at the hospital and dying while under home-based care were considered as poor outcomes [4.0% (12/303)] (Fig. 2).

### Determinants of treatment outcomes of COVID-19 patients under home-based care management in Kapelebyong district in eastern Uganda

Table 4 shows determinants of treatment outcomes of COVID-19 patients under home-based care. Age and time taken in care were determinants of treatment outcomes of COVID-19 patients managed under home-based care. Patients above 60 years of age had 17.4 times the odds of having poor treatment outcomes as those below 60 years old (Adjusted Odds Ratio (AOR): 17.4; 95% Confidence Interval (95% CI): 2.2–137.6). Patients who spent > 1 month in care had 15.3 times the odds of having poor treatment outcomes as those that spent less than 1 month in care (AOR: 15.3; 95% CI: 1.6–145.7).

**Table 4:** Description of COVID-19 patients managed under home-based care who died in Kapelebyong district in eastern Uganda

Age (years)	Sex	Name of facility patient was attached to	Date of death	Confirmed COVID-19 case	Enrolled into HBC	Co-morbidity	Vaccination status
54	Male	Acowa health center III	2021-09-26	Yes	Yes	Hypertension	Not vaccinated
69	Female	St.Francis Acumet health center III	2021-10-10	Yes	Yes	Hypertension	Not vaccinated

### Qualitative component

Our findings are organized into five main sections, experiences living with COVID-19 and experiences related to interaction with the health care settings, coping mechanism, interaction with people in the community, perception towards homecare management, and recommendations to improve COVID-19 home-based care (Table 5).

**Table 5:** Determinants of poor treatment outcomes among COVID-19 patients managed under home-based care in Kapelebyong district in eastern Uganda

Characteristic	Crude odds ratio (COR)	95% CI	*p*-value	Adjusted odds ratio (AOR)	95% CI	*p*-value
*Age*						
< 60	1.0			1.0		
≥ 60	44.5	8.9–221.5	< 0.001	17.4	2.2–137.6	0.007*
*Hypertension*						
No	1.0			1.0		
Yes	17.0	4.5–64.8	< 0.001	2.6	0.3–26.0	0.410
*Difficulty in breathing*						
No	1.0			1.0		
Yes	13.4	3.0–60.6	0.001	11.3	0.9–140	0.057
*Health worker follow-up*						
Yes	1.0			1.0		
No	0.2	0.03–0.8	0.024	0.3	0.03–2.6	0.272
*Time taken in care*						
< 2 weeks	1.0			1.0		
3 to 4 weeks	0.6	0.1–5.6	0.630	0.3	0.02–4.4	0.391
> 1 month	25.0	5.6–110.8	< 0.001	15.3	1.6–145.7	0.018*
*Side effects with drugs*						
No	1.0			1.0		
Yes	4.3	1.1–17.1	0.036	4.4	0.6–34.7	0.162

COR, Crude Odds Ratio; CI, Confidence interval; AOR, Adjusted Odds Ratio; *statistically significant at a *p*-value < 0.05

### Experiences living with COVID-19

#### Loss of economic and social life

Participants felt burdened by the cost of COVID-19 care. They argued that being at home without working during the isolation period brought loss of personal and family income. One participant noted that:

…*“even my business died. The little money I had, I ensured that I get some things” [P06, 37 years male, IDI]*

Another one then said,

…“There *was no where we would go to get money for food because we were just home” [P10, 36years, female, IDI*].

Additionally, there was also infringement on social life, as they argued that their social life was interrupted as they had been told to stay home to save others from getting COVID-19, as noted by this 36-year-old female,

…*“everything changed, imagine being at home for three weeks without doing anything. That affected my family financially. I couldn't even move to my friends. I was always home” [P10, 36years, female, IDI]*

#### Stigma associated with a COVID-19 patient label

Participants reported having experienced stigma as they sought services from the health facilities. They argued that the stigma was from health workers as well as from other people who had also gone to seek medical services from the health facilities. The stigma was in the form of health workers not attending to COVID-19 patients or suspects, other patients not willing to share wards with COVID-19 patients, and people not willing to associate with or be close to COVID-19 participants and people talking bad about those diagnosed with COVID-19 as these two participants narrated to us,

…“*Even fetching water and buying food from the market became a problem because people didnt want us to be close to them****”**** [P01, 35 years, female, IDI*]..

Another said,

“…*there's no way I could move anywhere because when people see you, they would think you're spreading the disease. Even the pupils I was teaching, they could see me and run. They even nicknamed, "corona". One day I tried to move out because they'd told me to be doing exercise, when the children saw them, they started shouting, "corona, corona" and we decided to come back home” …* [*P11;60 years, female, IDI*].

### Feeling isolated

The feeling of isolation was characterized the interaction that was there with friends, family and community members. They argued that other community members including their close friends didn’t want to associate or be in close contact with them because of their positive COVID-19 status due to fear of them acquiring COVID-19 disease. A 30-year-old male told us,

… “*one thing I can't forget is how my friends left me just because of Covid. I was really left out”* [*P03, 30 years male, IDI*].

As much as the participants faced isolation and stigma in different forms, even their caretakers had similar experience from the community as two participants explained,

*“In fact, every person who lived with me in the same compound was stigmatized every time they tried to go to the shops to buy something. Even when they went to the borehole, they would say that, "you people have COVID-19 at your place*"[*P08, 21years female, IDI]*. Participants also were denied a chance to buy items, one reported,

“*When I got money and I wanted to send someone for airtime, the mobile money person refused my money. The person said, "She has COVID-19. I can't touch her money" when I asked her to take money and use sanitizer, she said, "no, I can't take your money because you're sick" so that thing made me feel bad*” [*P09, 42years, female IDI*]

### Coping mechanism

The COVID-19 pandemic created a number of disruptions, fear, anxiety, uncertainty and economic loss to the patients. Our participants described a number of strategies they used to cope with challenges they encountered with COVID-19 and home-based care.

### Visits from friends and family

Participants felt better with other people visiting and interacting with them and as a 60-year-old female told us,

…“*Even when we were this sick, my father would come and greet us, "hey, how are you?" This would give us some hope including some friends. They would come and check on us and even bring firewood. It made me feel good knowing that some people care” [P11, 60 years, female from, IDI*]

### Engaging authorities

We found that some participants during their time in home care management; they were rejected and stopped from roaming around by the communities. The caretakers too were subjected to these same discriminatory feelings, as they were denied access to water and other things. In those cases, they had to engage the authorities to come and intervene. A participant told us,

…“*I had to call the health workers to talk to the LC1 because he was among the people who were telling people not to visit us. I think when the health worker talked to them, they understood and they stated letting us get water. We would take the jerrycan, they fill for us and then we would pick the water”. [P10, 36 years, female, IDI*]

### Use of preventive measures

Participants argued that they had to separate themselves from the family as a way of preventing other family members from getting infected since they had fear of infecting others. They also reported wearing masks and keeping social distance as a way of preventing other people from getting infected with COVID-19.

…“*we actually had to separate ourselves. Those who were sick stayed in a different room from those who were negative and we also never shared meals. We were wearing masks and keeping a distance from each other” [P11, 60years, female…IDI*]

### Praying to God

Participants prayed to God for healing and support during that difficult time when they were isolated at home. They believed it was only God who could heal them and save them from the suffering brought by COVID-19. One of them said,

*… “I prayed. I believed that God would heal me. I prayed not only once or twice but like over five times a day” [P01, 35 years, female …IDI]*

### Engaging in exercise

Others decided to keep themselves busy doing things that would keep them relaxed and busy like exercising. They reported that this was also part of the advice given to them by health workers, to do exercise while at home. One mentioned,

“*I was encouraged to do exercises like jogging but I would go to the garden because that's also vigorous exercise and I liked it. I also did jogging and gardening and that's what helped me recover faster. [Laughs] I knew that was a minor disease to me” [P01, 35years, female, IDI*]

### Use of complementary medicines

There was increased use of traditional and complementary medicines such as herbs, ginger, *nim* tree leaves, steaming and fruits. They reported that they were advised to do this by health workers to boost their immunity and help fight COVID-19. They said even their friends advised them to use herbs and steaming to quicken their recovery process at home.

*…“What I wouldn't forget was the first thing the doctors told me, they said that whichever thing I eat, I would have vegetables and fruits to boost my body strength to fight COVID-19” [P11, 60years, female …IDI]*

*… “I bought the drugs but also took some local herbs like nim tree leaves, I steamed, I also used ginger” [P06, 37 years, male …IDI]*

### Perception towards home-based care

Many participants believed being managed at home is better than being at the hospital. They argued that at home they were with family and friends and had freedom to run their family affairs even when isolated. They also reported that being at home kept them free from other hospital infections. One participant said.

…“*Home is better than me being treated from hospital because I may easily get another infection because there are so many people at the hospital who have other diseases besides COVID-19 that's why I feel like being at home is much better*” [*P01, 35years, female IDI*]].

Another participant said,

…“*I think it's good to be treated at home because you will have your family members that can support you, the only challenge I faced was with food because for me, I was isolated with my husband and other family members and so getting food was a challenge*” [*P10, 36years, female, …IDI*]. Another participant also said,

…“*home-based care was the best option for me I think because I also had time to be with my people even if I was sick because I would also look after them and monitor how they're doing things at home”* [*P03, 30years, male, IDI*].

### Recommendations to improve COVID-19 home-based care

Participants recommended several ways to improve their experiences managing COVID-19 from home, they also talked about alternative treatment besides what had been recommended by the medics, sensitization of masses to prevent stigma among others.

The need for sensitization of masses to equip them with knowledge on how to manage a COVID-19 patient was suggested by participants. One said,

…“*the gap was in sensitization. I think the community would have been sensitized about COVID-19 but maybe they wouldn't because the health workers are few and they could not do the sensitization fully. I think the negative attitude we got from the community was because of the ignorance of the community about COVID-19” [P11, 60years, female …IDI*]

Another suggested that treatment should have been provided at no cost. She said,

…“*COVID-19 is also a dangerous disease but why's it that people who have HIV are helped and are given free treatment unlike us who were using our own money to buy drugs*” [*P06,37years, male, …IDI*]

Some participants suggested that follow-up visits by health workers would help improve COVID-19 management at home;

*…“my thinking was, if the health workers continued visiting, maybe my neighbors and friends wouldn't have abandoned me” [P03, 30years, male, IDI] and a 26 year old female said, “What I wasn't happy about, since I was sent home, no health workers visited me. If they had been visiting, we would have improved faster”* [*P07, 26, female …IDI*]

## Discussion

In our study, almost all (96%) COVID-19 patients under home-based care had a good outcome. This could be because most of the cases managed at home were of milder COVID-19 [6]. Our findings are similar to those observed among home quarantined patients with COVID-19 in China, where 91.9% of COVID-19 patients had a good treatment outcome [5]. We also found out that the likelihood of a poor treatment outcome increased with age. This finding was not surprising. Other studies have also revealed that older patients were more likely to have poor treatment outcomes compared to the younger ones [7–10]. This could be related to the fact that the body’s immune defense system deteriorates with age hence older persons are more likely to develop severe disease and poor treatment outcomes from COVID-19 infection [11]. Our data also revealed that, patients who spent more than a month in home-based care had higher odds of poor treatment outcomes compared to those who spent less than a month in home-based care. This is similar to findings from a study conducted in china that also found out that patients who spent a longer time in home-based care had poor treatment outcomes [5]. Spending longer time in home-based care could signify existence of disease complications previously not detected.

### Experiences of COVID-19 patients managed under home-based care

Our findings show that COVID-19 patients managed under home-based care, experienced stigma and isolation. Our findings on stigma and isolation experienced by COVID-19 patients managed at home, can be explained using the theory of social stigma, as described by Erving Goffman [12]. Stigma is a phenomenon, whereby an individual with an attribute which is deeply discredited by their society is rejected as a result of the attribute [12]. Goffman described stigma as a process by which the reaction of others spoils normal identity [12]. In our findings having COVID-19 was an attribute discredited by the society in which these patients were managed hence they were stigmatized and isolated. This finding is in line with a study conducted in Pakistan that found that quarantined individuals were more likely to report stigmatization and social rejection [13].

The anxiety and fear surrounding COVID-19 disease was precipitated by the general awareness that COVID-19 is a deadly disease. A good number of participants expressed feeling of fear of developing severe disease or death. Various news outlets such as radio stations and television channels exacerbated the fear. For instance, high numbers of COVID-19 infections and mortalities in Europe and America were being reported and this could have contributed to the fear and anxiety our participants experienced [14]. The fear can also be understood using the death anxiety theory which states that many of people's daily behaviors consist of attempts to deny death and to keep their anxiety under strict regulation [15]. The death anxiety theory suggests that as an individual develops mortality salience, or becomes more aware of the inevitability of death, they will instinctively try to suppress it out of fear [15]. Our findings are in line with a study in southeast Ethiopia which found out that being an urban resident and access to news about COVID-19 disease severity and death increased the level of anxiety among COVID-19 patients by 16.8% [16].

Participants also suffered economic loss. The effects of isolation and restriction of movements throughout the entire treatment period led to loss of personal and family income in patients who were without any formal employment. Many participants alluded to losing their personal and family source of income due to their sickness. The economic loss experience was reported more among families that had both husband and wife (key bread earners) isolated. In comparison, COVID-19 management strategies have been associated with economic consequences worldwide [17]. For example, a study done in Uganda found that COVID-19 public health restrictions have a severe impact not only on older adults, but also on the whole family in Uganda [18]. Governmental strategies to contain the virus need to provide more support to enable people to get basic necessities and live as normal a life as possible.

We found that patients, who were believers, resorted to their faith as they tried to cope up with their sickness during their time in home-based care. This could be because most people believe that there is nothing impossible with God. Similarly, a study done in South Africa on spiritual care of COVID-19 patients found out that spiritual care is necessary to provide a means of coping and well-being for families, patients and health workers during the COVID-19 pandemic [19]. Getting support from the family members was also one of the most important coping strategies for patients under home-based care. They provide psychosocial support, food, drugs, performing activities of daily life, monitor patient’s health at home, encourage them to adhere to treatment and they contact health workers when need arises. This finding is in line with a study by Rahimi et al. 2021 where he reported that the family caregivers are reducing the increased burden of COVID-19 on health and social care systems [20]. As opposed to hospital care, where you are under the care of a nurse or a doctor, participants found being taken care of by a family member to be key to their treatment outcomes at home. This finding was in line with previous study conducted in the United Kingdom, where home-based care was compared to hospital-based care, which found home-based care to hold a slighter advantage in terms of recovery outcomes for older patients and surgical patients [21]. In this study, caregivers support was attributed to older patients’ recovery at home.

### Strengths and limitations

This was the first study, to the best of our knowledge, that investigated the treatment outcomes of COVID-19 patients managed under home-based care in rural Uganda, and it can be used to improve the quality of home-based care in the country. We conducted a mixed-methods study, combining both quantitative and qualitative methods of data collection. The use of the two approaches in this study increases the rigor, trustworthiness and angles at which we investigated the outcomes. The qualitative findings helped us make meaning of the quantitative results. For instance, the in-depth interviews allowed us to capture participants’ lived experiences under home-based care. To ensure trustworthiness in this study, we ensured that after transcribing the transcript and analyzing data, the results were read to a few participants for validation. The use of two coders also helped us to increase the coding rigor and credibility of our results. Since we used the district register of all patients managed at home, and included 303 participants out of a population of 309 patients in this list, we believe selection bias could be minimal in our study. Since we investigated hard outcomes such as death and hospitalization, misclassification of the outcome is probably minimal in the study. We also visited the patients at their homes and this reduced the potential of selection bias. However, it is possible, that some patients did not present to any health facility, and hence our results, apply to only patients who got COVID-19 and presented to a health facility. We could have increased this rigor by verifying with hospital records and village death registers but unfortunately this was not done. For information bias, we used a standardized questionnaire for all participants and for qualitative part, probing questions were used.

Our study sample size (number of outcomes) was small for determining factors associated with poor outcomes, and this can be seen by the wide confidence intervals of our results. As such it is possible that our study was not powered to determine some factors associated with poor outcomes. Hence, some associations may not have been observed due to lack of power.

Additionally, the knowledge that the principal investigator in this study was the district focal person for home-based care services in Kapelebyong and played a key role in coordinating COVID-19 home-based care activities, could have also biased the way participants were recruited and responded to our questions. However, the inclusion of almost all the patients managed under home-based care (303) out of the population of (309) patients, could have reduced this selection bias anticipated to distort the results of this study.

## Conclusions

Majority of the patients had a good treatment outcome. Age above 60 years and long stay in home-based care were associated with poor treatment outcome. Stigma, fear, anxiety, rejection, not being followed up by health workers and economic loss were identified as negative experiences encountered during home care. Our study supports previous recommendations of community sensitization on COVID-19 and home-based care to address stigma and rejection and more systematic follow up of patients under home-based care by health workers. We recommend more research on the safety and effectives of home-based care in resource limited settings.

## Data Availability

The datasets used and/or analyzed during the current study are available from the corresponding author on reasonable request.

## Abbreviations

CHW: Community Health worker
COVID-19: Corona virus disease 2019
DHO: District Health Officer
HIV/AIDS: Human immunodeficiency virus/acquired immunodeficiency syndrome
IPC: Infection Prevention and Control
MoH: Ministry of Health
PCR: Polymerized chain reaction
PPE: Personal protective equipment
SARS-CoV-2: Severe acute respiratory syndrome corona virus type 2
VHT: Village Health Team
W.H.O: World Health Organization
